# Intratumoral recombinant human interferon alpha‐2a and vincristine combination therapy in canine transmissible venereal tumour

**DOI:** 10.1002/vms3.119

**Published:** 2018-08-17

**Authors:** Halit Kanca, Gizem Tez, Kazim Bal, Dogukan Ozen, Eray Alcigir, Sevil Atalay Vural

**Affiliations:** ^1^ Department of Obstetrics and Gynaecology Ankara University Ankara Turkey; ^2^ Department of Biostatistics Faculty of Veterinary Medicine Ankara University Ankara Turkey; ^3^ Department of Pathology Faculty of Veterinary Medicine Ankara University Ankara Turkey

**Keywords:** Canine, immunochemotherapy, recombinant human interferon *α*‐2a, transmissible venereal tumour, vincristine

## Abstract

Canine transmissible venereal tumour (CTVT) is a naturally occurring contagious neoplasm of dogs located mainly on the external genitalia of both sexes. The course of vincristine chemotherapy, the most effective and practical therapy, is affected by the immune status of the host. The aim was to investigate recombinant human interferon alpha‐2a (rhIFN *α*‐2a) and vincristine for treatment of CTVT. A total of 21 female dogs were included. In group I (*n* = 9), vincristine (0.025 mg/kg, IV) was administered weekly. In group II (*n* = 6), dogs were injected intratumorally weekly with 1.5 million IU rhIFN *α*‐2a. In group III (*n* = 6), rhIFN *α*‐2a and vincristine were combined. No tumour regression was observed after three injections of rhIFN *α*‐2a in group II and weekly vincristine was administered. The number of tumour infiltrating lymphocytes (TILs), mitotic figures and apoptotic cells were counted in subsequent incisional tumour biopsies. The Kaplan–Meier Method was used to analyse survival using complete tumour regression as the outcome and Breslow Test was used for comparison of survival curves. Differences in TILs, cell proliferation and apoptosis between groups were assessed by analysis of covariance. Complete regression was observed in all animals included. Mean duration of vincristine treatment for complete regression was shorter in group II (3.50 weeks, 95% CI, 3.06–3.94, *P* < 0.05) and group III (3.17 weeks, 95% CI, 2.84–3.49, *P* < 0.01) compared to group I (5.11 weeks, 95% CI, 4.42–5.80). Vincristine and rhIFN *α*‐2a combination increased TILs in CTVT biopsies compared to vincristine treatment (*P* = 0.017) and vincristine treatment after rhIFN *α*‐2a (*P* = 0.049). Vincristine treatment after rhIFN *α*‐2a (Group II;* P* < 0.001) and rhIFN *α*‐2a and vincristine combination (Group III;* P* < 0.001) decreased apoptosis. The results indicate that intratumoral rhIFN *α*‐2a treatment alone is not effective in CTVT. However, combination of rhIFN *α*‐2a and vincristine shortens the duration of treatment compared to vincristine therapy.

## Introduction

Canine transmissible venereal tumour (CTVT) is a naturally occurring contagious neoplasm of dogs located mainly on the external genitalia of both sexes (Cohen 1985). CTVT is usually transmitted during coitus and it is endemic in more than 90 countries across all inhabited continents (Strakova & Murchison 2014). Although spontaneous regression can occur, CTVTs are usually progressive and are treated accordingly. Vincristine chemotherapy is the most effective and practical therapy (Martins *et al*. 2005). Vincristine is used weekly at a dose of 0.025 mg/kg, intravenously (IV). Complete regression is usually achieved after 2–8 injections and the cure rate is over 90% (Calvert *et al*. 1982; Nak *et al*. 2005).

Spontaneous and experimentally induced CTVT shows an initial progressive growth, followed by a brief static period and then regression (Gonzalez *et al*. 2000). Host immune response against the tumour was suggested to affect the course of spontaneous tumour regression (Trail & Yang 1985; Mizuno *et al*. 1989); however, studies concerning immunotherapy are sparse. In addition, the immune status of the host was suggested to play role during vincristine chemotherapy. Both spontaneous and vincristine‐induced tumour regressions are characterized by an increased number of tumour infiltrating lymphocytes (TILs). Vincristine chemotherapy decreases cell proliferation in tumour tissue and apoptosis is observed (Scarpelli *et al*. 2010).

In human medicine, recombinant human interferon alpha‐2a (rhIFN*α*‐2a) has been exploited in both single‐agent and multimodality protocols to treat numerous neoplastic conditions. Human IFN*α* has been shown to affect canine lymphocyte functions and enhance both interleukin (IL)‐2 production and natural killer (NK) cell cytotoxicity in dogs (Krakowka *et al*. 1988). In canine medicine, recombinant interferon has been used primarily for its antiviral activity and for its immunomodulatory properties in the treatment of immune‐mediated or infectious ophthalmological and dermatological conditions (Gilger *et al*. 1999; De Mari *et al*. 2003; Stokking *et al*. 2004; Thompson *et al*. 2004). In addition, it offers a therapeutic option for canine epitheliotropic lymphoma (Tzannes *et al*. 2008). We therefore investigated rhIFN*α*‐2a and vincristine for treatment of CTVT.

## Materials and methods

Animal experimentation was approved by the respective local authority (Local Ethics Committee on Animal Experiments, Ankara University, Turkey; Approval no: 2012‐5‐37).

Twenty‐one female dogs from different breeds with progressing natural CTVT at external genitalia which were referred to the Clinic of Obstetrics and Gynaecology, Faculty of Veterinary Medicine, Ankara University, Turkey, were used. The mean age and body weight of dogs were 43.6 ± 25.12 months and 25.6 ± 10.24 kg, respectively. Clinical diagnosis was confirmed by vaginal cytology. A cotton collection swab was gently placed on the lesion and rotated. After rolling on a polylysine‐coated slide, the smear was stained following the Papanicolaou technique as described. CTVT cells were identified as large, round cells containing eosinophilic, distinctly vacuolated cytoplasm, and round nuclei with coarse chromatin and one to two large nucleoli (Erunal Maral *et al*. 2000). Dogs were housed in standard laboratory conditions for an acclimatisation period of 2 weeks and the tumours were regarded as progressing based on increasing size during acclimatisation period. Three‐view thoracic radiographs were taken and abdominal ultrasonography was performed in order to exclude animals with distant metastasis.

The dogs were divided into 3 groups and the average sizes of tumours in each group was similar. In group I (*n* = 9), intravenous vincristine (Atafarm) was administered weekly at a dose of 0.025 mg/kg. In group II (*n* = 6), the dogs were injected intratumorally weekly with 1.5 million IU (5.55 mcg/0.25 mL) rhIFN*α*‐2a (Roferon^®^ Roche). In group III (*n* = 6), rhIFN*α*‐2a and vincristine were combined. All dogs were examined clinically once a week. Complete blood counts were performed at each clinical visit prior to the administration of chemotherapeutic agents. Cutoff values of 100 000 cells *μ*L^−1^ for thrombocyte count, 1500 cells *μ*L^−1^ for neutrophil count and <30% packed cell volume (PCV) were used when deciding whether or not therapy would be administered (Finlay *et al*. 2017). Side effects were recorded and graded as described by Veterinary Cooperative Oncology Group (Veterinary Cooperative Oncology Group, 2004). Complete disappearance of the lesion and absence of CTVT cells on vaginal smears was considered a complete response to treatment. Because no tumour regression was observed after three injections of rhIFN*α*‐2a in group II, the animals received weekly vincristine administrations. Mean duration of vincristine treatment for complete regression was recorded in all groups.

At admission and immediately before each treatment, tumour volume was measured and incisional biopsies were taken until complete tumour regression. Tumour volume was estimated using the standard ellipsoid formula: length × width × height × *π*/6 as described (Scarpelli *et al*. 2010). In multifocal disease, tumour volume was assessed by measuring only the largest tumour focus. The subsequent biopsies were immersion fixed in 4% neutral formaldehyde and processed routinely. Haematoxylin and eosin‐stained paraffin sections were evaluated. The average number of TILs and mitotic figures were attained by counting cells in 10 × 400 high power field under light microscope. For the quantitative analyses of apoptosis in haematoxylin and eosin‐stained paraffin sections, terminal deoxynucleotidyl transferase‐mediated dUTP‐FITC nick end‐labelling (TUNEL) assay was used (Roche Diagnostics) according to instructions. The median number of TUNEL‐positive cells were obtained by counting cells in 10 × 400 high power field under light microscope. Canine thymus tissue sections served as positive control (Mukaratirwa *et al*. 2009).

Statistical analyses were performed with the statistical software SPSS 14.01 (SPSS Inc, Chicago, IL). Data are presented as mean values ± standard error (SE). To analyse time‐related effects of intralesional rhIFN*α*‐2a on TILs, cell proliferation, apoptosis and tumour volume; the assumptions of normality and homogeneity of variance of the data were tested using the Shapiro–Wilks test and the Levene's test, respectively. For data that assumptions were observed, repeated measures analysis of variance was used to test overall statistical significance. For tumour volume data, the assumptions were not observed and Friedman repeated measures analysis of variance on ranks was used. *P* ˂ 0.05 was considered to be statistically significant.

All analyses were based on intent to treat and included all subjects for treatment groups. The Kaplan–Meier Method was used to analyse survival, using complete tumour regression as the outcome and Breslow Test was used for comparison of survival curves.

Differences in TILs, cell proliferation and apoptosis between treatment groups were assessed by analysis of covariance (ANCOVA) using change from initial to end point as the dependent variable, with treatment as fixed effect and the corresponding pretreatment values as covariate. The difference in mean change from pretreatment values between treatment groups was estimated using adjusted mean values along with the associated SE and 95% CI from the ANCOVA model. The study was designed to provide 90% power to detect an average difference of 0.5% in TILs, mitotic figures and apoptosis between treatment groups.

## Results

Recombinant human IFN*α*‐2a therapy alone did not result in tumour regression (Group II). Average tumour volume after three injections of rhIFN*α*‐2a was not different from average tumour volume at the beginning of the therapy (*P* *>* 0.05). rhIFN*α*‐2a therapy was discontinued and the animals in this group received vincristine therapy accordingly. The effects of intralesional rhIFN*α*‐2a injections in CTVT are illustrated in Table 1. The number of TILs in CTVT biopsies was not affected by rhIFN*α*‐2a injections (*P* *>* 0.05). In contrast, the numerical values of mitotic figures (*P* ˂ 0.05) and apoptotic cells (*P* ˂ 0.01) were decreased.

**Table 1:** Effects of intralesional rhIFN*α*‐2a injections in CTVT (Mean ± SEM)

Parameters	Before treatment	Week 1	Week 2	Week 3	*P*
TILs	6.43 ± 0.84	6.15 ± 0.63	6.85 ± 0.68	6.37 ± 0.41	˃0.05
Mitotic figures	2.00 ± 0.54^a^	0.88 ± 0.35^b^	0.45 ± 0.23^bc^	0.45 ± 0.32^bc^	˂0.05
Apoptotic cells	10.28 ± 0.70^a^	9.52 ± 1.29^ab^	8.53 ± 1.11^b^	7.15 ± 1.00^b^	˂0.01
Tumour volume (cm^3^)	36.94 ± 6.64	41.28 ± 7.42	39.25 ± 6.41	36.42 ± 5.55	˃0.05

Within columns, values with different superscripts (a, b, c) are significantly different.

**Figure 1:**
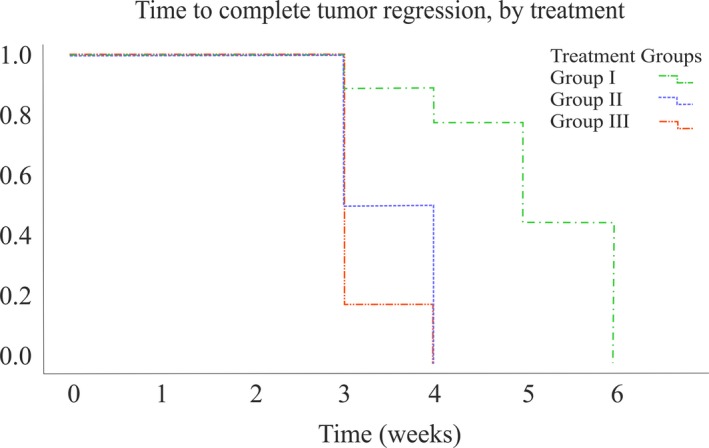
Kaplan–Meier curve of time to complete tumour regression of dogs that received vincristine (group I), vincristine after rhIFN *α*‐2a (group II) and vincristine and rhIFN *α*‐2a combination (group III).

Complete regression was observed in all animals included in the study. Mean duration of vincristine treatment for complete regression (Fig. 1) was shorter in group II (3.50 weeks, 95% CI, 3.06–3.94, *P* < 0.05) and group III (3.17 weeks, 95% CI, 2.84–3.49, *P* < 0.01) compared to group I (5.11 weeks, 95% CI, 4.42–5.80). No recurrences were observed in dogs after a period of 2–18 months.

Average numbers of TILs, mitotic figures and apoptotic cells in CTVT biopsies during vincristine therapy in study groups are given in Table 2.

**Table 2:** Average number of tumour infiltrating lympocytes (TILs), mitotic figures, apoptotic cells and tumour volume during vincristine therapy (Group I), vincristine therapy after rhIFN*α*‐2a injections (Group II) and rhIFN*α*‐2a and vincristine combination therapy (Group III) (Mean ± SEM)

Groups		Before 1st treatment	Before 2nd treatment	Before 3th treatment	Before 4th treatment	Before 5th treatment
Group I		*n* = 9	*n* = 9	*n* = 8	*n* = 7	*n* = 4
	TILs	6.03 ± 2.81	7.72 ± 3.14	10.86 ± 3.32	13.04 ± 2.90	14.23 ± 3.82
	Mitotic figures	2.57 ± 1.55	1.68 ± 1.02	0.95 ± 0.84	0.33 ± 0.33	0.30 ± 0.14
	Apoptotic cells	9.29 ± 5.27	14.54 ± 6.79	14.88 ± 6.97	16.17 ± 3.67	19.38 ± 6.60
	Tumour volume (cm^3^)	31.41 ± 2.91	19.17 ± 2.21	9.19 ± 1.29	1.69 ± 0.19	0.41 ± 0.02
Group II		*n* = 6	*n* = 6	*n* = 3	–	–
	TILs	6.37 ± 1.00	12.92 ± 4.04	17.03 ± 1.76	–	–
	Mitotic figures	0.45 ± 0.78	0.83 ± 0.13	0.07 ± 0.12	–	–
	Apoptotic cells	7.15 ± 2.46	4.40 ± 3.17	1.10 ± 0.98	–	–
	Tumour volume (cm^3^)	36.42 ± 5.55	10.82 ± 2.08	2.31 ± 0.49	–	–
Group III		*n* = 6	*n* = 6	*n* = 1	–	–
	TILs	6.60 ± 1.88	23.65 ± 16.79	11.3	–	–
	Mitotic figures	2.22 ± 1.64	0.52 ± 0.86	–	–	–
	Apoptotic cells	10.08 ± 3.42	3.30 ± 2.21	0.60	–	–
	Tumour volume (cm^3^)	37.73 ± 5.94	13.80 ± 3.26	6.85 ± 0.00	–	–

There was a significant effect of treatment groups on levels of last TIL measurements after controlling for the effect of first TIL measurements. Planned contrasts revealed that treating animals with rhIFN*α*‐2a and vincristine (Group III), significantly increased the number of TILs in CTVT biopsies compared to vincristine treatment (Group I; *P* = 0.017) and vincristine treatment after rhIFN*α*‐2a (Group II; *P* = 0.049). The covariate (first mitotic figure measurement) significantly predicts the last mitotic figure measurement. Therefore, the last mitotic figure measurement is influenced by the first mitotic figure measurement (*P* = 0.047). There was no significant effect of treatment groups on levels of last mitotic figure measurements after controlling for the effect of first mitotic figure measurements. There was a significant effect of treatment groups on levels of last apoptosis measurements after controlling the effect of first apoptosis measurements. Planned contrasts revealed that treating animals with vincristine (Group I), significantly increased the number of apoptotic cells compared to vincristine treatment after rhIFN*α*‐2a treatment (Group II; *P* < 0.001) and rhIFN*α*‐2a and vincristine combination (Group III; *P* < 0.001). Summary of ANCOVA showing the effect of treatment groups on the number of TILs, mitotic figures and apoptotic cells are illustrated in Table 3.

**Table 3:** Summary of ANCOVA showing the effect of treatment groups on the number of tumour infiltrating cells, mitotic figures and apoptotic cells (Mean ± SEM)

Treatment group	Tumour infiltrating cells	Mitotic figures	Apoptotic cells
Group I	12.20 ± 2.91^a,^ a	0.24 ± 0.20^b^	16.52 ± 1.19^a,^ c
Group II	14.20 ± 3.56^a,^ a	0.53 ± 0.24^b^	2.39 ± 1.46^b,^ c
Group III	24.36 ± 3.56^b,^ a	0.53 ± 0.24^b^	2.30 ± 1.46^b,^ c

ANCOVA = analysis of covariance. Within column values with different superscripts (a, b) are significantly different (*P* < 0.05).

a Covariates appearing in the model are evaluated at the following values: TILs Time 1 = 6.3095.

b Covariates appearing in the model are evaluated at the following values: Mitotic figures Time 1 = 2.3048.

c Covariates appearing in the model are evaluated at the following values: Apoptotic cells Time 1 = 9.8000.

Hematological side effects observed were mild and transient (Grade I) not requiring suspension of one or more chemotherapeutic administrations. Thrombocytopenia was observed in 3, 1 and 2 dogs in groups I, II and III, respectively. Neutropenia was observed in 1 dog in each study groups. Decreased PCV values were observed in 2 dogs in group I and in 1 dog in group III. Gastrointestinal effects observed were Grade I anorexia, vomiting and diarrhoea requiring no medical intervention. Anorexia (2/9), vomiting (1/9) and diarrhea (2/9) were observed in vincristine group. Diarrhoea (1/6) was the only gastrointestinal side effect observed in group II. One case for each gastrointestinal side effect was observed in group III.

## Discussion

The results show that rhIFN*α*‐2a immunotherapy alone is not effective in CTVT regression as tumour volume is not affected. In addition, the number of TILs in tumour tissue is not affected by rhIFN*α*‐2a injections. Increasing lymphocyte infitration, particularly T‐lymphocytes, has been associated with transition from progression to regression phase (Trail & Yang 1985; Mizuno *et al*. 1994; Mukaratirwa & Gruys 2003). Both humoral and cellular immunities are important for CTVT regression and different immunmodulators have been used in CTVT (Mukaratirwa *et al*. 2009; Hess *et al*. 1977; Den Otter *et al*. 1999). Results from immunotherapy studies; however, have been considered unsatisfactory compared to chemotherapy (Purohit 2008). However, a relatively long time is needed for tumour regression is another limiting factor for practical use of immunotherapy in CTVT (Den Otter *et al*. 1999; Den Otter *et al*. 2015). Although we observed a decrease in cell proliferation and apoptosis, rhIFN*α*‐2a therapy was stopped after 3 injections and long‐term effects of intralesional rhIFN*α*‐2a immunotherapy was not assessed.

Vincristine therapy resulted in complete regression in all animals. Considering inclusion criteria that only animals bearing progressing tumours without distant metastasis were used, high cure rates were expected. Cure rates approach 100% when vincristine is used in progressive tumours, particularly in early cases (Boscos & Ververidis 2004). Complete regression in all animals studied enabled us to use Kaplan–Meier Method to analyse survival using complete tumour regression as the outcome.

Despite a limited number of study cases, this study shows that rhIFN*α*‐2a and vincristine immunochemotherapy of CTVT shortens the duration of treatment compared to vincristine therapy alone. Minimizing cost and side effects related to chemotherapy is of importance for practical reasons. During vincristine therapy involution of the lesions is gradual, requiring clinic visits for each application (Calvert *et al*. 1982; Nak *et al*. 2005). A higher number of vincristine injections increases concern for chemosafety issues. In the current study routine, weekly vincristine administrations were made and animals were clinically examined before each administration. Considering the significant reduction in the number of vincristine administrations during immunochemotherapy, it would be interesting to observe CTVT regression after smaller number of immunochemotherapy applications for durations longer than 1 week.

Vincristine‐induced CTVT regression is characterized by increases in the number of TILs (Gonzalez *et al*. 2000). A significant increase is observed in the number of TILs during rhIFN*α*‐2a and vincristine immunochemotherapy that may at least partly explain the shortening in the duration of treatment compared to vincristine therapy alone. According to the current model of the interaction of CTVT with host immune cells, tumour cells lack Major Histocompatibility Complex (MHC) molecules and release transforming growth factor‐*β* (TGF‐ *β*), which suppresses T cells and NK cells during the growth phase (Siddle & Kaufman 2014). Although TILs produce high levels of IFN‐*γ* which promotes MHC expression, IFN‐*γ* activity is inhibited by TGF‐*β* (Hsiao *et al*. 2008). However, during regression, TILs produce high concentrations of IL‐6, antagonizing TGF‐*β* (Hsiao *et al*. 2004). The levels of IFN‐*γ* and MHC expression increase, leading to cytotoxicity by T cells and NK cells (Siddle & Kaufman 2014). Little is known on the effects of rhIFN*α*‐2a on CTVT. In one report, human IFN*α* has been shown to affect various canine lymphocyte functions and enhance both IL‐2 production and NK cytotoxicity in dogs (Krakowka *et al*. 1988).

Vincristine sulphate, a vinca alkaloid mitosis inhibitor, binds to *β*‐tubulin at specific binding sites, interfering with microtubule formation. It destroys the mitotic spindle of the cell and prevents further cell division in the G1 phase (Van Vuuren *et al*. 2015). Mitotic figures ceased in all groups studied which is in agreement with previous reports (Gonzalez *et al*. 2000; Mukaratirwa *et al*. 2009). Interestingly, intralesional rhIFN*α*‐2a injections without vincristine significantly reduced the number of mitotic figures, indicating a antiproliferative effect of rhIFN*α*‐2a in CTVT. Inhibition of cell proliferation is one of the main anticancer effects of IFN‐*α* (Caraglia *et al*. 2013).

Researchers using vincristine for CTVT treatment described high percentages of apoptosis as a mechanism of tumour regression (Gonzalez *et al*. 2000; Stockmann *et al*. 2011). Vincristine treatment increased apoptosis confirming previous reports. In contrast, a decrease in the number of apoptotic cells were observed when vincristine was used after or in combination with rhIFN*α*‐2a injections. Furthermore, intralesional rhIFN*α*‐2a injections without vincristine reduced apoptosis as well. Concomitant reduction in the number of apoptotic cells with fast CTVT regression rate seems contradictory. In addition to inhibiting cell proliferation and/or suppressing oncogene expression, IFN‐*α* exerts its anticancer effects by promoting cell apoptosis (Parker *et al*. 2016) and it has often been used as a sensitizing agent for the treatment of various human malignancies (Shi *et al*. 2016). Furthermore, intracystic injections of IFN‐*α* in human cystic craniopharyngiomas were shown to reduce the tumour by activating the Fas apoptotic pathway (Ierardi *et al*. 2007). The reason for reduced apoptosis observed is not known. The number of apoptotic cells in CTVT biopsies may have been confounded due to sampling time. Tumour biopsies were taken weekly before each vincristine injections in the current study, which means that the effect on apoptosis was determined 1 week after each treatment. On the other hand, the distribution half‐life _(t1/2*α*, 21.5 min)_ of vincristine in dogs is relatively short, the uptake of the drug by tubulin‐rich tissues is high (Hantrakul *et al*. 2014) and CTVT regression is initially very fast. Furthermore, cells undergoing prolonged arrest of mitosis by cytotoxic drugs generally go into a typical process of apoptosis from the beginning; or pass to a dropout process of ‘slippage’, in which cells depart from mitosis and return to the G1 phase (Topham & Taylor 2013).

Gastrointestinal disorders, hypo‐ or hypertension, tachycardia, headache and resistance development are the main side effects of IFN‐*α* therapy in humans (Foser *et al*. 2006). Interferons are anecdotally used in some canine malignancies, though randomized clinical trials are lacking. Several veterinary trials evaluating IFN‐*α* in combination with standard chemotherapy are being conducted (Regan & Dow 2015). The main disadvantages of interferon use in dogs are cost and need for daily injections. Resistance due to antibody production against interferons has not yet been proved in dogs (Clifford *et al*. 2000). Mild and transient haemotological and gastrointestinal side effects observed are in agreement with previous reports, using vincristine for CTVT treatment (Calvert *et al*. 1982; Nak *et al*. 2005). Side effects observed did not require suspension of drug administrations, not only in the vincristine group but also in the study groups in which rhIFN*α*‐2a was used. Accordingly, the weekly administration frequency and intralesional route of rhIFN*α*‐2a seems to be safe in dogs with CTVT.

The results of the current study indicate that intratumoral rhIFN*α*‐2a treatment alone is not effective in treatment of CTVT. However, a combination of rhIFN*α*‐2a and vincristine shortens the duration of treatment compared to vincristine therapy alone, which is promising from a practical point of view.

## Source of funding

This research was not funded by a specific project grant.

## Conflicts of interest

The authors declare no potential conflict of interest.

## Ethical statement

The authors confirm that the ethical policies of the journal, as noted on the journal's author guidelines page, have been adhered to and the appropriate ethical review committee approval has been received.

## Contributions

Study design: Halit Kanca. Clinical work: Gizem Tez and Kazim Bal. Statistical analysis: Dogukan Ozen. Pathological assessment: Eray Alcigir and Sevil Atalay Vural. Draft manuscript preparation: Halit Kanca and Gizem Tez. Manuscript revision and approval: Halit Kanca and Gizem Tez.
